# Analysis of common type 2 diabetes mellitus genetic risk factors in new-onset diabetes after transplantation in kidney transplant patients medicated with tacrolimus

**DOI:** 10.1007/s00228-012-1292-8

**Published:** 2012-05-09

**Authors:** Mateusz Kurzawski, Krzysztof Dziewanowski, Joanna Łapczuk, Anna Wajda, Marek Droździk

**Affiliations:** 1 Department of Experimental and Clinical Pharmacology, Pomeranian Medical University, Powstancow Wlkp. 72, 70-111 Szczecin, Poland; 2 Clinical Department of Nephrology and Dialysis, Marie Curie Regional Hospital, Arkonska 4, 71-455 Szczecin, Poland

**Keywords:** NODAT, Kidney transplantation, Single nucleotide polymorphisms, Type 2 diabetes mellitus

## Abstract

**Purpose:**

New-onset diabetes after transplantation (NODAT) is a major complication after kidney transplantation. The risk factors for NODAT include the use of calcineurin inhibitors as part of the immunosuppressive regimen, among which tacrolimus has the most pronounced diabetogenic effect. Both NODAT and type 2 diabetes mellitus (T2DM) share several risk factors. Recent studies have identified a number of common genetic variants associated with increased risk of T2DM. Here we report the results of our study on the potential effect of single nucleotide polymorphisms (SNPs) previously associated with T2DM on the risk of NODAT in kidney transplant patients medicated with tacrolimus.

**Methods:**

Seven SNPs in six genes known to increase the risk of T2DM in Caucasians were genotyped by means of TaqMan assays in 235 kidney transplant patients medicated with tacrolimus: rs4402960 and rs1470579 in *IGF2BP2*; rs1111875 in *HHEX*; rs10811661 upstream of *CDKN2A/B*; rs13266634 in *SLC30A8*; rs1801282 in *PPARG*; rs5215 in *KCNJ11*. The *TCF7L2* rs7903146 SNP was also included in the multivariate analysis.

**Results:**

None of the analyzed SNPs was significantly associated with the risk of NODAT. However, the *IGF2BP2* rs4402960 T allele was present significantly more frequently among patients diagnosed with NODAT more than 2 weeks after transplantation (*p* = 0.048). Mean (± standard deviation) number of the analyzed alleles tended to be lower in patients without NODAT (6.19 ± 1.71) than in NODAT patients (6.58 ± 1.1.95; *p* = 0.09) and significantly lower compared to late-onset NODAT patients (7.03 ± 1.88; *p* = 0.018). Multivariate analysis confirmed the significance of ‘diabetogenic’ allele number in late-onset NODAT development [odds ratio (OR) 1.37, 95 % confidence interval (CI) 1.05–1.78; *p* = 0.017]. Additionally, individuals carrying >7 of the analyzed ‘diabetogenic’ alleles were at a significantly higher risk of NODAT (OR 2.17, 95 % CI 1.18–3.99; *p* = 0.015).

**Conclusions:**

Complex analysis of genotypes increasing the risk of diabetes may lead to the identification of NODAT susceptibility predictors.

## Introduction

New-onset diabetes after transplantation (NODAT) is one of the major complications after kidney transplantation. The term ‘NODAT’ has replaced the older term ‘post-transplant diabetes mellitus’ (PTDM) to differentiate new-onset diabetes from diabetes developed prior to transplantation [1]. It is now widely accepted that NODAT leads to graft failure and promotes cardiovascular disease, the main cause of death in kidney transplant recipients [2]. The incidence of NODAT in patients after solid organ transplantation reported in a large meta-analysis ranged from 2 to 50 % at 1-year post-transplantation [3]. The pathophysiology of NODAT closely mimics that of type 2 diabetes mellitus (T2DM), with both diseases characterized by a combination of insulin resistance and insulin hyposecretion. However, insulin hyposecretion has been a key determinant of worsening glucose tolerance following renal transplantation [2]. The risk factors for NODAT include both the conventional risk factors for T2DM [e.g., older recipient age, nonwhite ethnicity, family history of diabetes, sedentary lifestyle, high body mass index (BMI), and cytomegalovirus or hepatitis C virus (HCV) infection] and those specific to transplant patients (acute rejection incidence, high doses of corticosteroids, and an immunosuppressive regimen with calcineurin inhibitors) [4, 5].

Among the calcineurin inhibitors used in transplant recipients, the diabetogenic effect of tacrolimus seems to be the most pronounced. In one study, patients receiving tacrolimus had a significantly higher incidence of NODAT than those not medicated with the drug [4]. In another study, patients treated with tacrolimus had a significantly higher incidence of NODAT or impaired fasting glucose after 6 months of therapy than those treated with cyclosporine (33.4 vs. 26.0 %, respectively [6]. Tacrolimus has been reported to cause NODAT through decreased insulin secretion of pancreatic beta cells in an animal model, with inhibition of insulin mRNA transcription [7]. Decreased insulin release as a consequence of high tacrolimus concentration was confirmed in human studies; however, insulin resistance was also suggested as a possible mechanism [8, 9]. An elevated risk of developing NODAT has also been described in prediabetic patients [9]. Hence, NODAT is most probably not a separate entity but a consequence of an underlying glucose metabolic disorder that is uncovered by immunosuppression [5].

Past linkage studies have identified those genes associated with the most prominent T2DM effects, i.e., *TCFL7*, *PPARG*, and *KCNJ11*. More recently genome-wide association studies (GWAS) have confirmed previous reports and uncovered dozens of new genetic variants associated with an increased risk of T2DM [10–15]. A number of the single nucleotide polymorphisms (SNPs) identified to be associated with T2DM are common in Caucasians and may underlie individual susceptibility to NODAT. In some cases an association of T2DM risk alleles with NODAT has been confirmed. A polymorphism of the *KCNQ1* gene, which encodes the pore-forming subunit of the voltage-gated K+ channel, has been associated with an increased risk for NODAT in Spanish Caucasians [16], independently of other risk factors. Variants of hepatocyte nuclear family transcription factor 4 alpha (*HNF4A*) and insulin receptor substrate-1 (*IRS1*) have been associated with NODAT among Hispanic American renal allograft recipients, who are known to have a higher risk of NODAT compared to other Caucasians, despite early steroid withdrawal [17]. An influence of SNPs within the calpain-10 gene (*CAPN10*) on the occurrence of diabetes in Polish Caucasian kidney transplant recipients has also been described [18]. Additionally, several diabetogenic alleles have been reported to be correlated with NODAT risk in Koreans [19].

The aim of the study reported here was to analyze the potential effect of SNPs previously associated with T2DM on NODAT development in kidney transplant patients medicated with tacrolimus. For the purpose of this study, seven SNPs in six genes, with the frequency of minor allele being >0.1 in Caucasians, which had been previously identified and confirmed as ‘diabetogenic’ in the general population in GWAS studies were selected: rs4402960 and rs1470579 in the *IGF2BP2* intronic region, rs1111875 in near *HHEX*, rs10811661 upstream of *CDKN2A/B*, rs13266634 missense in *SLC30A8*, rs1801282 in the *PPARG* intron, and rs5215 missense in *KCNJ11*.

## Methods

### Patients

A total of 235 kidney transplant patients, all Polish Caucasians and non-diabetic at the moment of transplantation (patients with diabetes mellitus prior to the transplantation were excluded) were eligible for enrollment in this retrospective study. Subjects were recruited consecutively from patients who underwent kidney transplantation in the Clinical Department of Nephrology and Dialysis, County Hospital, Szczecin, Poland between 2000 and 2009 and who were subsequently medicated with twice-daily tacrolimus (Prograf; Astellas Pharma, Tokyo, Japan) as a part of immunosuppressive regimen. Patients who did not maintain graft function for at least 1 year post-transplant were excluded. The patients eligible for enrollment were subdivided into two groups: those with NODAT (*n* = 67) and the controls, i.e., without NODAT (*n* = 168). The characteristics of the patients are given in Table 1. Patients with hemoglobin A1c continuously >6.5 mg/dL, fasting plasma glucose of >126 mg/dL (7.0 nmol/L), or those requiring insulin and/or oral hypoglycemic agents for >3 months were diagnosed as having NODAT. NODAT was diagnosed up to 1 year post-transplantation. The observation time was extended in the case of some late-onset NODAT patients in order to achieve the 3-month evaluation period from the onset of diabetes. The NODAT patients were subsequently divided into two groups: those with early-onset NODAT (*n* = 39), in whom diabetes occurred within the first 2 weeks of immunosuppressive therapy, and those with late-onset NODAT, in whom diabetes was found later in the course of treatment (*n* = 28).

**Table 1:** Characteristics of patients enrolled in the study

Patient characteristics	NODAT (*n* = 67)	No PTDM (*n* = 168)	*p*
Age (years)	47.7 ± 10.6	43.2 ± 13.0	0.014^b^
Sex (female)	30 (45.5)	78 (46.4)	1.000^c^
Body mass index	25.8 ± 4.1	24.3 ± 3.7	0.006^b^
Donor age (years)	47.8 ± 11.8	46.0 ± 12.3	0.321^b^
Viral infections^a^	10 (15.2)	27 (16.1)	1.000^c^
Acute rejection	9 (13.6)	10 (6.0)	0.064^c^
Steroid total dose (g)	4.20 ± 1.72	3.76 ± 2.35	0.017^d^

NODAT, New-onset diabetes after transplantation; PTDM, post-transplant diabetes mellitus

Data are presented as the mean ± standard deviation, or as the number, with the percentage in parenthesis

^a^Viral infections were: cytomegalovirus (6 vs. 20 in PTDM and control group, respectively), hepatitis C virus (2 vs. 4), and hepatitis B virus (2 vs. 3)

^b^Student *t* test

^c^Fisher exact test

^d^Mann–Whitney *U* test

The treatment protocol consisted of tacrolimus, mycophenolate mofetil, and steroids. Specifically, tacrolimus therapy was initiated at 0.1 mg/kg/day with doses adjusted to maintain trough levels of between 10 and 12 ng/mL in the first month post-transplantation, and then between 8 and 10 ng/mL. Whole blood tacrolimus concentration was assessed with the use of a chemiluminescent microparticle immunoassay (CMIA; Architect Tacrolimus Assay, Abbott, Germany). An initial oral dose of mycophenolate mofetil 2.0 g/day was administered once daily or given in equally divided doses every 12 h. Methylprednisolone was given concomitantly: a dose of 500 mg on the day of surgery, tapered to 40 mg/day during the first week, followed by 30 mg/day of prednisolone in the second week, 20 mg/day of prednisolone in the third week, 15 mg/day in the fourth week, and 10 mg/day thereafter. Total corticosteroid dose for each patient during the first year of the study was calculated, and methylprednisolone was recalculated to prednisolone using *r* = 1.25 cofactor. All patients gave written informed consent to participate in the study, and a relevant ethics committee approved the study protocol.

### Genotyping

Genomic DNA was extracted from 200 μL of whole blood samples using the GeneMATRIX Quick Blood DNA Purification kit (EURx, Gdansk, Poland). Pre-validated allelic discrimination TaqMan real-time PCR assays (Applied Biosystems, Foster City, CA) were used to detect SNPs previously associated with T2DM in the general population. The following SNPs in their respective genes were analyzed (assay IDs are given in parentheses): rs4402960 (C___2165199_10) and rs1470579 (C___2165184_10) in *IGF2BP2*, rs1111875 (C__11214581_10) in *HHEX*, rs10811661 (C__31288917_10) in *CDKN2A*, rs13266634 (C____357888_10) in *SLC30A8*, rs1801282 (C___1129864_10) in *PPARG*, and rs5215 (C___2991148_10) in *KCNJ11*. Fluorescence data were captured using an ABI PRISM 7500 FAST Real-Time PCR System (Applied Biosystems) after 40 cycles of PCR.

### Statistical analysis

Categorical variables (i.e., allele, genotype, haplotype frequencies, acute rejection episodes) were compared by the Fisher exact test and chi-square test. Odds ratios (OR) and 95 % confidence intervals (95 % CI) were calculated using the Newcombe–Wilson method without the continuity correction. Multivariate logistic regression model was used to test independent NODAT risk factors. Variables identified as potentially significant (*p* < 0.1) were included in multivariate analysis (age, BMI, acute rejection episodes, total steroid dose) together with the sum of alleles previously associated with increased risk of T2DM in the general population: *IGF2BP2* rs4402960 T, *HHEX* rs1111875T*, CDKN2A* rs10811661 C*, SLC30A8* rs13266634 C, *PPARG* rs1801282 C, and *KCNJ11* rs5215 C. The *TCF7L2* rs7903146 T allele was added to the multivariate analysis, as genotyping data were available from our previous study for all patients [20] and this SNP has also been associated with T2DM [21]. *IGF2BP2* rs1470579 C was not further analyzed, as it was found to be in almost complete linkage disequilibrium with *IGF2BP2* rs4402960 T (*D*′ = 1.0, *r* ^2^ = 0.981), forming a common haplotype that includes minor alleles for both loci. Hence, it could not be considered as an independent variable. In each patient, the number of ‘diabetogenic’ alleles was calculated for a given SNP (i.e., 0, 1, or 2), and values for all SNPs were subsequently summarized to obtain a total number of ‘diabetogenic’ alleles. A *p* level of <0.05 was considered to be statistically significant. The data were tested for their fit to Hardy–Weinberg equilibrium by calculating expected frequencies of genotypes and comparing them to the observed values using a chi-square test. All calculations were performed using the Statistica 9.0 software package (Statsoft, Tulsa, OK).

## Results

Mean patient’s age, BMI, and total steroid dose were higher in the NODAT group than in the non-NODAT patients (controls). No differences in gender, donor age, viral infection frequency, nor acute rejection episodes were noted between these two groups (Table 1). In terms of genotype frequency distribution, none of the SNPs analyzed showed a significant deviation from the Hardy–Weinberg equilibrium.

**Fig. 1:**
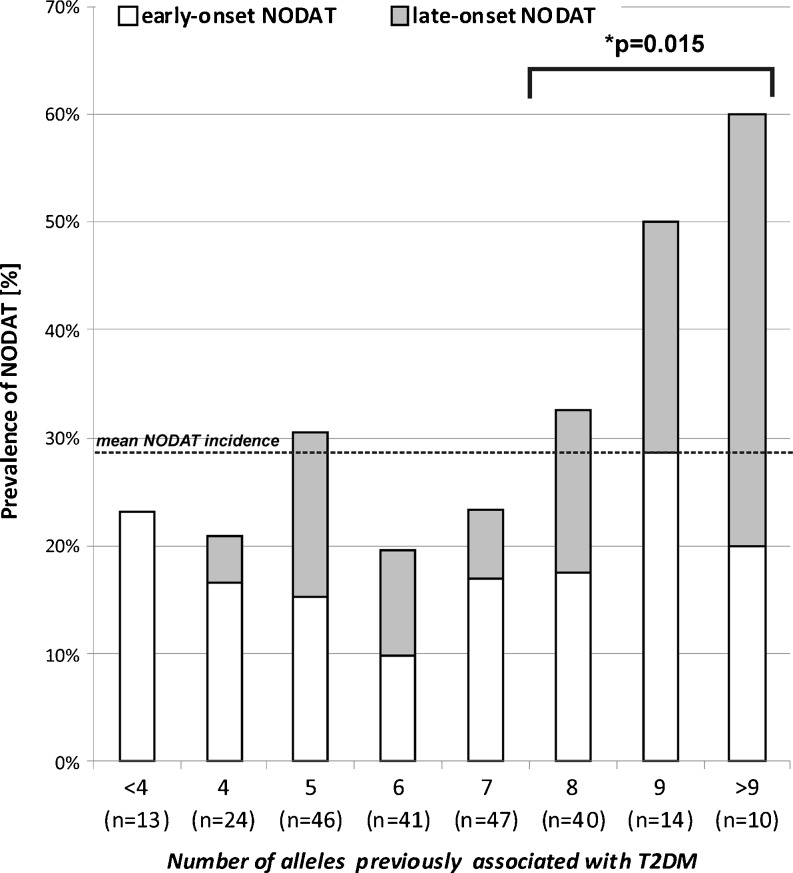
Incidence of new-onset diabetes after transplantation (*NODAT*) in relation to the sum the alleles previously associated with type 2 diabetes mellitus (*T2DM*) (*IGF2BP2* rs4402960 T, *HHEX* rs1111875 C, *CDKN2A/B* rs10811661 T, *SLC30A8* rs13266634 C, *PPARG* rs1801282 C, *KCNJ11* rs5215 C, and *TCF7L2* rs7903146 T). *Asterisk* indicates significance between patients inheriting ≤7 vs. >7 alleles in relation to overall NODAT incidence, calculated by means of Fisher exact test (odds ratio 2.17, 95 % confidence interval 1.18–3.99)

None of the analyzed SNPs was significantly associated with the risk of NODAT (Table 2). However, of the seven SNPs analyzed, six (with the exception for *KCNJ11* rs5215) alleles previously identified as T2DM risk factors occurred more frequently in the NODAT group than in patients who did not develop diabetes (difference was not significant). The NODAT patients were subsequently divided into those with early-onset NODAT (*n* = 39) and late-onset NODAT (*n* = 28), respectively. No association between genetic factors and early-onset NODAT was observed, although the *IGF2BP2* rs4402960 T allele was found significantly more frequently among patients diagnosed with NODAT later than 2 weeks post-transplantation (*p* = 0.048; Table 2). Of the seven analyzed ‘diabetogenic’ alleles, six were more frequent in the late-onset NODAT patients than in the patients without diabetes (exception: the *PPARG* rs1801282 C allele). Due to the small number of patients in the subgroups, these differences were not significant. In order to investigate a cumulative effect of SNPs previously associated with T2DM in relation to NODAT, we analyzed the total number of ‘diabetogenic’ alleles inherited by each patient. The mean number of analyzed alleles tended to be lower in patients without NODAT (6.19 ± 1.71) than in all NODAT patients (6.58 ± 1.1.95; *p* = 0.09) and to be significantly reduced in comparison with late-onset NODAT patients (7.03 ± 1.88; *p* = 0.018). Multivariate analysis confirmed the significance of ‘diabetogenic’ allele number in the risk of developing late-onset NODAT (OR 1.37, 95 % CI:1.05–1.78; *p* = 0.017), which was also influenced by the occurrence of graft rejection episodes (Table 3). The sum of alleles was not associated with early-onset NODAT (OR 1.03, 95 % CI 0.83–1.27; *p* = 0.801), rather, steroid total dose was identified as a main independent risk factor. Additionally, when patients were classified according to the number of alleles previously associated with T2DM, those carrying more than a half of ‘diabetogenic’ alleles (>7 of the 14 alleles within 7 genes) were at significantly higher risk of NODAT (OR 2.17, 95 % CI 1.18–3.99; *p* = 0.015; Fig. 1).

**Table 2:** The genotype and allele frequencies in patients with NODAT compared to patients without NODAT

Single nucleotide polymorphism	Without NODAT (*n* = 168)		NODAT (all) (*n* = 67)				Early-onset NODAT (*n* = 39)				Late-onset NODAT (*n* = 28)			
	*n*	%	*n*	%	*p*	OR (95 % CI)	*n*	%	*p*	OR (95 % CI)	*n*	%	*p*	OR (95 % CI)
*IGF2BP2* rs4402960														
GG	79	47.0	26	38.8	0.309^b^	0.71 (0.40–1.27)	18	46.2	1.000^b^	0.96 (0.48–1.94)	8	28.6	0.099^b^	0.45 (0.19–1.08)
GT	69	41.1	33	49.3	0.281	1.45 (0.79–2.67)	19	48.7	0.713	0.83 (0.40–1.70)	14	50.0	0.172	2.00 (0.79–5.06)
TT	20	11.9	8	11.9	0.807	1.21 (0.48–3.01)	2	(5.1	0.159	0.29 (0.06–1.33)	6	21.4	0.086	2.96 (0.92–9.51)
Allele														
G	227	67.6	85	63.4			55	70.5			30	53.6		
T^a^	109	32.4	49	36.6	0.389		23	29.5	0.685		26	46.4	0.048	
*IGF2BP2* rs1470579														
AA	77	45.8	26	38.8	0.383^b^	0.75 (0.42–1.34)	18	46.2	1.000^b^	1.01 (0.50–2.04)	8	28.6	0.101^b^	0.47 (0.20–1.13)
AC	71	42.3	33	49.3	0.356	1.37 (0.75–2.52)	19	48.7	0.717	1.14 (0.56–2.35)	14	50.0	0.253	1.90 (0.75–4.79)
CC	20	11.9	8	11.9	0.808	1.18 (0.47–3.01)	2	5.1	0.357	0.43 (0.99–1.99)	6	21.4	0.090	2.89 (0.90–9.28)
allele:														
A	225	67.0	85	63.4			55	70.5			30	53.6		
C^a^	111	33.0	49	36.6	0.517		23	29.5	0.593		26	46.4	0.068	
*HHEX* rs1111875														
TT	61	36.3	19	28.4	0.287^b^	0.69 (0.37–1.29)	12	30.8^b^	0.579	0.78 (0.37–1.65)	7	25.0	0.289^b^	0.58 (0.23–1.45)
CT	76	45.2	40	59.7	0.116	1.69 (0.88–3.21)	22	56.4	0.439	1.47 (0.67–3.20)	18	64.3	0.185	2.06 (0.81–5.26)
CC	31	18.5	8	11.9	0.817	0.83 (0.33–2.10)	5	(12.8	1.000	0.82 (0.27–2.54)	3	10.7	1.000	0.84 (0.20–3.49)
Allele														
T	198	58.9	78	58.2			46	59.0			32	57.1		
C^a^	138	41.1	56	41.8	0.917		32	41.0	1.000		24	42.9	0.883	
*CDKN2A/B* rs10811661														
TT	121	72.0	53	79.1	0.323^b^	1.47 (0.75–2.89)	30	76.9	0.689^b^	1.29 (0.57–2.93)	23	82.1	0.356^b^	1.78 (0.54–4.97)
CT	45	26.8	14	20.9	0.405	0.71 (0.36–1.40)	9	23.1	0.689	0.81 (0.35–1.83)	5	17.9	0.358	0.58 (0.21–1.63)
CC	2	1.2	0	0.0	1.000	-	0	0.0)	1.000	-	0	0.0	1.000	-
Allele														
T^a^	287	85.4	120	89.6			69	88.5			51	91.1		
C	49	14.6	14	10.4	0.294		9	11.5	0.588		5	8.9	0.301	
*SLC30A8* rs13266634														
CC	73	43.5	4	6.0	0.448^b^	0.60 (0.19–1.87)	19	48.7	0.594^b^	1.23 (0.61–2.48)	14	50.0	0.543^b^	1.30 (0.58–2.89)
CT	79	47.0	30	44.8	0.589	1.52 (0.47–4.91)	17	43.6	0.711	0.83 (0.40–1.71)	13	46.4	0.835	0.86 (0.38–1.94)
TT	16	9.5	33	49.3	0.425	1.81 (0.56–5.82)	3	7.7	0.760	0.72 (0.19–2.73)	1	3.6	0.455	0.32 (0.04–2.66)
Allele														
C^a^	225	67.0	38	28.4			55	70.5			41	73.2		
T	111	33.0	96	71.6	0.379		23	29.5	0.593		15	26.8	0.439	
*PPARG* rs1801282														
CC	125	74.4	52	77.6	0.737^b^	1.19 (0.61–2.33)	31	79.5	0.543^b^	1.33 (0.57–3.12)	21	75.0	1.000^b^	1.03 (0.41–2.59)
CG	39	23.2	15	22.4	0.865	0.92 (0.47–1.82)	8	20.5	0.833	0.83 (0.35–1.94)	7	25.0	1.000	1.06 (0.42–2.70)
GG	4	2.4	0	0.0	0.580	-	0	0.0	1.000		0	0.0	1.000	-
Allele														
C^a^	289	86.0	119	88.8			70	89.7			49	87.5		
G	47	14.0	15	11.2	0.454		8	10.3	0.461		7	12.5	1.000	
*KCNJ11* rs5215														
TT	58	34.5	27	40.3	0.453^b^	1.28 (0.71–2.29)	17	43.6	0.355^b^	1.46 (0.72–2.97)	10	35.7	1.000^b^	1.05 (0.46–2.43)
TC	80	47.6	27	40.3	0.336	0.72 (0.38–1.36)	17	43.6	0.443	0.72 (0.34–1.54)	10	35.7	0.629	0.73 (0.28–1.85)
CC	30	17.9	13	19.4	1.000	0.93 (0.42–2.06)	5	12.8	0.443	0.57 (0.19–1.69)	8	28.6	0.428	1.55 (0.55–4.32)
Allele														
T	196	58.3	81	60.4			51	65.4	0.305		30	53.6	0.559	
C^a^	140	41.7	53	39.6	0.755		27	34.6			26	46.4		

CI, Confidence interval; OR, odds ratio

Significance determined with the Fisher exact test, compared to patients without NODAT, calculated using homozygotes for a major allele/major allele count as reference, with the exception of those marked with superscript 'b'

^a^‘Diabetogenic’ alleles

^b^Fisher exact test, calculated using homozygotes for a major allele vs. minor allele carriers

**Table 3:** Multivariate logistic regression analysis of potential risk factors for early-onset and late-onset NODAT

Independent variables	Early NODAT (*n* = 39)		Late NODAT (*n* = 28)	
	OR (95 % CI)	*p*	OR (95 % CI)	*p*
Patient’s age	1.02 (0.99–1.05)	0.181	1.02 (0.99–1.06)	0.206
BMI (kg/m^2^)	1.05 (0.95–1.16)	0.315	1.11 (0.99–1.24)	0.074
Acute rejection episodes	0.76 (0.17–3.40)	0.724	5.47 (1.55–19.22)	0.008
Steroid total dose^a^	2.40 (1.05–5.51)	0.037	0.93 (0.50–1.72)	0.801
Sum of the risk alleles^b^	1.03 (0.83–1.27)	0.801	1.37 (1.05–1.78)	0.017

Early NODAT, up to 14 days post-transplantation; late-onset NODAT, developed later than 14 days post-transplantation

^a^Logarithmic transformation applied to fit normal distribution

^b^Sum of alleles previously associated with T2DM: *IGF2BP2* rs4402960 T, *HHEX* rs1111875 C, *CDKN2A/B* rs10811661 T, *SLC30A8* rs13266634 C, *PPARG* rs1801282 C, *KCNJ11* rs5215 C, and *TCF7L2* rs7903146 T

## Discussion

Both NODAT and T2DM share several risk factors (i.e., overweight or obesity, recipient age, Hispanic and African-American ethnicity, CMV or HCV infection, age >40 years). Genetic factors may also play an important role, since a family history of T2DM among first-degree relatives has been identified as a NODAT risk factor [4]. In our study, we analyzed the potential effect of SNPs previously associated with T2DM on NODAT development in kidney transplant patients medicated with tacrolimus. An association between the SNPs investigated and T2DM was definitely confirmed, but their effect was relatively mild, with the odds ratios for most variants ranging from 1.10 to 1.20, with a maximum of about 1.4 for *TCF7L2* rs7903146 T. *TCF7L2* rs7903146 T is the most prominent of all T2DM-associated common polymorphisms identified to date [13, 22]. It is clear that one cannot detect the magnitude of the effects of these polymorphisms without analyzing thousands of patients. However, as the incidence of diabetes among transplant recipients medicated with tacrolimus is much higher than that in the general population, we had actually expected the influence of the investigated genetic variants on NODAT risk to be much more pronounced, as found by Yang et al. who observed an adjusted odds ratio value of ≥2 for SNPs within *HNF4A* and *IRS1* genes among Hispanic American kidney transplant recipients [17]. A study of Korean patients confirmed the association of eight SNPs in six genes (i.e., *TCF7L2*, *SLC30A8*, *HHEX*, *CDKAL1*, *CDKN2A/B*, and *KCNQ1*) with NODAT in subjects medicated according to different immunosuppressive regimens (NODAT developed at different times during the observation period, with a median follow-up of >10 years) in a relatively large cohort of kidney transplant recipients. However, a long observation time might lead to an overlap of NODAT and T2DM in a later post-transplant period. The significance of analyzed variants could not be confirmed in our study, possibly due to the different ethnicity of the study participants, but also partly due to the limited number of patients analyzed, which is certainly one of the limitations of our study. The number of participants was limited as only patients receiving tacrolimus were included. Tacrolimus is known to increase the risk of NODAT in kidney allograft recipients compared to other immunosuppressants [4, 6]. The majority of studies performed to date on the association of genetic factors with NODAT have included all patients regardless of immunosuppressive regimen [17, 19, 23]. In this respect, the group analyzed in our study is more homogenous, as all study participants were treated according to the same regimen (tacrolimus + mycofelate mofetil + steroids), which may translate into an increased sensitivity of the analysis.

Our investigation of the risk factors for early-onset NODAT (developed in the first 2 weeks post-transplantation) and late-onset NODAT revealed significant differences between these subgroups of patients. Total steroid dose, including methylprednisone administered as an induction of immunosuppressive therapy, significantly increased the risk of diabetes in the former group (early-onset) but not in the latter one. Indeed, mean steroid dose in the first week post-transplantation was significantly higher in early-onset NODAT patients (35.3 ± 6.9 mg/day prednisolone) than in patients without NODAT (31.5 ± 8.5 mg/day prednisolone; *p* = 0.017) as well as to the late-onset NODAT group (31.3 ± 7.7 mg/day prednisolone; *p* = 0.031). This observation may indicate that early-onset NODAT is triggered rather by steroids, while late-onset NODAT is probably more dependent on other risk factors. The frequency of acute rejection episodes and total steroid dose are known to be associated with PTDM and are usually linked since high methylprednisolone doses (pulse therapy) are administered in the case of graft rejection. In our study, multivariate analysis revealed the occurrence of acute rejection episodes as an independent late-onset NODAT risk factor, while steroid dose was the only adjusted risk factor of early-onset NODAT. However, some patients received monoclonal antibodies instead of steroids during an acute rejection episode, which may explain the observed differences.

In order to investigate the cumulative effect of SNPs previously associated with T2DM on NODAT occurrence, we assessed the total number of ‘diabetogenic’ alleles inherited by each patient. Summing up the alleles of different genes would seem to be an acceptable approach since the effect of each SNP on T2DM risk is comparable [13, 22, 24, 25]. Our analysis revealed that individuals carrying more than half of the total possible number of ‘diabetogenic’ alleles (>7 of the 14 alleles of 7 genes) were at a significantly higher risk of NODAT, primarily due to an increased risk of late-onset NODAT in patients inheriting a greater number of ‘risk’ alleles. These findings were also confirmed in the multivariate analysis, where the sum of ‘diabetogenic’ alleles was revealed as a significant risk factor for late-onset—but not early-onset—NODAT. Based on the results of our study, we conclude that the effect of common polymorphisms in *IGF2BP2*, *HHEX*, *CDKN2A/B*, *SLC30A8*, *PPARG*, and *KCNJ11*, all of which have been previously associated with T2DM, is not pronounced in the risk of NODAT in kidney transplant recipients treated with tacrolimus. However, the sum of ‘diabetogenic’ alleles may be one of the factors increasing the incidence of post-transplant diabetes, especially in the case of late-onset NODAT, which seems to be less dependent on steroid treatment. Our results support the observation that a complex analysis of diabetes risk genotypes may lead to the identification of disease susceptibility predictors, also for NODAT, and that this approach may have an advantage over single-locus studies [26]. Finally, the application of the analyzed genetic markers in therapy individualization should be verified by further independent studies in populations of different ethnicities.
